# Akzeptanz und Nutzungsbedingungen digitaler Interventionen zur Distressprävention bei Studierenden: Ergebnisse aus einer qualitativen Interviewstudie entlang der „unified theory of acceptance and use of technology“ (UTAUT2)

**DOI:** 10.1007/s11553-022-00985-7

**Published:** 2022-10-06

**Authors:** Anne Krümmel, Isabella Laiker, Kamil J. Wrona, Leona Aschentrup, Christoph Dockweiler

**Affiliations:** 1 Fakultät für Gesundheitswissenschaften, Universität Bielefeld, Bielefeld, NRW Deutschland; 2 Fachbereich Ingenieurswissenschaften und Mathematik, Fachhochschule Bielefeld, Bielefeld, NRW Deutschland; 3 Fachbereich Gesundheit, Fachhochschule Bielefeld, Bielefeld, NRW Deutschland; 4 Lebenswissenschaftliche Fakultät, Universität Siegen, Siegen, NRW Deutschland

**Keywords:** Stressprävention, Stressmanagement, Digitale Gesundheit, UTAUT 2, E‑Mental-Health, Stress prevention, Stress management, Digital health, UTAUT 2, E‑mental-health

## Abstract

**Hintergrund:**

Das ausbildungsbezogene Stressempfinden auf Ebene von Distress von Studierenden stellt ein hohes Risiko für die Entstehung von psychischen Erkrankungen dar. Die konsequente Nutzung digitaler Anti-Stress-Apps kann dazu beitragen, Versorgungsdefizite in der Vermeidung von stressinduzierten Erkrankungen wirksam auszugleichen, wenn existierende Hilfsangebote nicht genutzt werden, oder helfen, Barrieren zur Nutzung bestehender Interventionsmaßnahmen mindern. In diesem Kontext untersucht der vorliegende Beitrag die Einstellungsakzeptanz und die verbundenen Nutzungsbedingungen Studierender der Universität Bielefeld gegenüber digitalen Anti-Stress-Apps. Basierend auf der Akzeptanz und den Nutzungsbedingungen sollen Anforderungen für die bessere Implementierung digitaler Interventionen geleitet werden.

**Methodik:**

Die Erhebung wurde mittels qualitativer leitfadengestützter Interviews und quantitativer Umfragen an denselben 15 Studierenden (*n* = 14 Frauen und einem Mann im Alter von 22 bis 31 Jahren) der Universität Bielefeld durchgeführt. Für die Auswertung der Interviews wurde die strukturierte Inhaltsanalyse nach Mayring herangezogen, bei der mit Hilfe von deduktiven und induktiven Kategorien die Transkripte analysiert wurden. Zur Strukturierung und Analyse der Ergebnisse wurde die „unified theory of acceptance and use of technology 2“ (UTAUT 2) eingesetzt.

**Ergebnisse:**

Anhand der UTAUT 2 konnten bedürfnisorientierte Bedingungen zur Nutzung und Einstellung hinsichtlich der Akzeptanz identifiziert werden. Die Einstellungsakzeptanz von Studierenden zu Anti-Stress-Apps ist weitestgehend positiv, geht jedoch nicht einher mit dem Nutzungsverhalten. Die Ergebnisse weisen eine starke Abhängigkeit von individuellen Bedürfnissen auf, was zudem von verschiedenen Alltagssituationen ausgeht, in denen Stress individuell empfunden wird.

**Schlussfolgerungen:**

Bedingungen an die vorhandenen Elemente und Interventionen in einer App zur Stressreduktion müssen in der Softwareentwicklung berücksichtigt werden, damit die Anwendung subjektiv als wirksam wahrgenommen wird. Der Ausbau und die Einführung bedürfnisorientierter Angebote in die Lebenswelt der Nutzer*innen und Nutzergruppen sowie begleitende Maßnahmen zur zielgruppenspezifischen Sensibilisierung, können die Akzeptanz weiter steigern, das Nutzungsverhalten positiv beeinflussen und gegen Stressbelastungen wirken.

## Abstract (en)

**Background:**

Educational distress perceived by students represents a high risk for the development of mental illness. Consistent use of digital antistress apps can effectively address and therefore compensate for care deficits in the prevention of stress-induced mental illnesses. In addition, consequent use can reduce barriers to the use of existing offers. Against this background, the acceptance and conditions regarding use of digital antistress apps by students at Bielefeld University were examined. Based on the acceptance and the conditions of use, requirements for better implementation of digital interventions are identified.

**Methods:**

Semi-structured interviews and quantitative surveys were used to conduct the study among the same 15 students (*n* = 14 women and 1 man, aged 22–31 years) at Bielefeld University. Mayring’s structured content analysis was used to evaluate the interviews, using deductive and inductive categories to analyze the transcripts. The unified theory of acceptance and use of technology 2 (UTAUT 2) was used to structure and analyze the results.

**Results:**

Using the UTAUT 2, crucial conditions regarding acceptance, use, and attitude were identified. The attitudinal acceptance of students towards antistress apps is largely positive, though this is not associated with usage behavior. Furthermore, our research reveals a strong dependence on individual needs, which is dependent on the individual daily situations and aspects in which stress is experienced.

**Conclusion:**

Conditions on the existing elements and interventions in an app for stress reduction must be considered in software development so that its use is subjectively perceived as effective. The development of needs-oriented offers and introduction into the lifeworld of the users as well as accompanying measures for target group-specific sensitization can further increase the acceptance, positively influence use and work against stress.

Distress stellt für Studierende eine große, alltägliche Belastung dar. Seit Jahren nehmen die psychischen Belastungen in dieser Lebensphase zu. Gleichzeitig bieten neue Technologien Betroffenen eine niedrigschwellige Option zum selbstständigen Stressmanagement. Damit Studierende primäre Stress-Apps nutzen, ist es notwendig, Faktoren, welche die Akzeptanz und Nutzungsbedingungen beeinflussen, zu identifizieren. Der vorliegende Beitrag untersucht anhand der UTAUT 2-Theorie („unified theory of acceptance and use of technology 2“) die Einstellungsakzeptanz von stressreduzierenden Apps bei Studierenden.

## Hintergrund

Innerhalb des jungen Erwachsenenalters kann das Studium als Lebensabschnitt überwiegend guter Gesundheit dennoch mit zentralen Entwicklungsherausforderungen charakterisiert werden. Umbrüche in den sozialen Beziehungen, zunehmende Autonomieanforderungen bei gleichzeitiger Verdichtung von Arbeitsprozessen in einer stark regelgeleiteten und vorstrukturierten Studienumgebung gehen allerdings auch mit erhöhten Prüfungsanforderungen u. a. aufgrund der Einführung von Bachelor‑/Masterstudiengängen im Zuge des Bologna-Prozesses sowie Versagensängsten oder Leistungsdruck innerhalb einer verkürzten Studiendauer einher [8, 11]. So gibt jeder zweite Studierende an, regelmäßig unter ausbildungsbedingtem Distress zu leiden, jeder dritte sogar dauerhaft. Fast die Hälfte der Studierenden fühlt sich dadurch erschöpft, begleitet von physischen Beeinträchtigungen wie z. B. Spannungskopfschmerzen oder allgemeines Unwohlsein. Dabei bewerten Studentinnen ihren Gesundheitszustand insgesamt schlechter als ihre männlichen Kommilitonen [9, 19]. Gleichzeitig werden Hilfsangebote wie z. B. psychologische oder psychotherapeutische Beratungsstellen und Hausärzt*innen von Studierenden nur wenig genutzt. Typische Barrieren sind lange Wartezeiten, geringe Flexibilität der Angebote, fehlende Motivation, die Infragestellung der Wirksamkeit und der Wunsch, die Probleme eigenständig lösen zu wollen [4, 14]. Vor diesem Hintergrund ist es notwendig, die Handlungsspielräume in der Prävention psychischer Belastungen bei Studierenden zielgruppenadäquat durch digitale Anwendungen zu erweitern [16, 21]. Darüber hinaus hat die COVID-19-Pandemie („coronavirus disease 2019“) zu einer verstärkten Stressbelastung bei Studierenden geführt. Im Rahmen einer Studie an der Ostfalia-Hochschule in Braunschweig, Wolfenbüttel und Wolfsburg nahmen 39,6 % der Teilnehmenden die Studiensituation während der Pandemie als stressiger wahr. Dabei sind u. a. Faktoren wie der Verlust oder die Stundenreduktion der Nebenbeschäftigung, Einschränkungen der sozialen Kontakte oder die Umstellung auf das digitale Lernen zusätzlich wahrgenommene Stressoren der befragten Studierenden [5]. Auch im internationalen Raum lassen sich ähnliche Tendenzen beobachten. So konnte bspw. eine Studie einen Anstieg des empfundenen Stresslevels bei Studierenden der Opole University of Technology im Süden Polens während der Coronapandemie feststellen [18]. Eine weitere Studie, welche die das wahrgenommene Stressniveau aufgrund des COVID-19-Ausbruchs an der University of Lorraine und der Sciences Po College in Frankreich untersuchte, konnte bspw. die Verschiebung einer Abschlussprüfung oder auch einen Rückgang der Lernzeit als assoziierende Faktoren mit einem hohen Stresslevel nachweisen. Auch Schwierigkeiten sich zuhause zu isolieren oder störende Geräusche hatten einen negativen Einfluss auf das Stresslevel der Studierenden [1]. Daher leidet insbesondere die psychische Gesundheit unter der Pandemie [2, 10].

Bei Studierenden aber können digitale Anwendungen aufgrund eines niederschwelligen Zugangs und einer Kongruenz zur selbstverständlichen Mediatisierung der Lebensgewohnheiten junger Erwachsener einen wichtigen Baustein in der Reduktion von Stressbelastung darstellen [13, 14]. Die Apps (Applikationen [Apps] bzw. Anti-Stress-Apps) zur Förderung der psychischen Gesundheit sind auf mobilen Endgeräten verfügbar und können autonom zeit- und ortsunabhängig genutzt werden und dienen Studierenden zum Selbstmanagement belastender Alltagssituationen [21]. Dabei wird zwischen primären Anti-Stress-Apps, die direkt die Erkrankungen oder spezifische Symptome fokussieren sowie sekundären Anti-Stress-Apps, die sich mehr auf die Reduktion von Risikofaktoren konzentrieren, unterschieden [7]. Obwohl das Interesse für die Nutzung von Anti-Stress-Apps vorhanden ist, nehmen die Studierenden diese nur begrenzt in Anspruch: Die Befragungen von Kern et al. (2018) fanden heraus, dass von insgesamt 741 Studierenden 26,1 % bereit E‑Mental-Health-Angebote nutzten, während 73,9 % diese vielleicht oder gar nicht nutzen würden, was folglich darauf hinweist, dass die Mehrheit eine Nutzung solcher Apps ablehnt [16]. Bisher haben erst wenige Studien die Akzeptanz von Apps zur Stressreduktion bei Studierenden untersucht [13]. Dennoch konnte herausgestellt werden, dass die Studierenden i. Allg. positiv gegenüber Anti-Stress-Apps eingestellt sind, aber der Meinung sind, diese nicht zu benötigen, da keine psychischen Beeinträchtigungen vorhanden sind. Zudem ist entsprechendes Marketing und die Bekanntmachung der Verfügbarkeit von Campuslizenzen notwendig, um solche Apps bei Studierenden präsenter zu machen und dadurch Nutzungsbedingungen zu verbessern und aktives Nutzungsverhalten zu fördern [10]. Insbesondere in der Forschung der DACH-Region findet noch keine ausreichende Beachtung international gebräuchlicher Operationalisierungen von Einstellungsakzeptanz statt. Vor diesem Hintergrund greift die vorliegende Studie auf die UTAUT 2 zurück. Mit dem Modell der UTAUT 2 werden die Technikakzeptanz und die Nutzungsbedingungen sowie das Nutzungsverhalten in Bezug auf Technik von Personen untersucht. Das Modell ist in die Bereiche Leistungserwartung, Aufwandserwartung, sozialer Einfluss, erleichternde Rahmenbedingungen, Preis-Leistung, Gewohnheit und hedonistische Motivation kategorisiert [20]. Durch die Verwendung der UTAUT 2 sollen die Einstellungsakzeptanz und Nutzungsbedingungen von Studierenden in Bezug auf Anti-Stress-Apps identifiziert werden.

Auf Basis dieses hier dargestellten Desiderats ergeben sich folgende handlungsleitende Forschungsfragen:

- Wie werden die Dimensionen der Einstellungsakzeptanz von primären Stress-Apps bei Studierenden, angelehnt an die theoretischen Variablen der UTAUT 2, wahrgenommen?
- Welche personenbezogenen Unterschiede (Soziodemographie, Technikaffinität, Belastungssituation) zeigen sich in der Einstellungsakzeptanz von primären Stress-Apps bei Studierenden (angelehnt an die Variablen der UTAUT 2)?
- Welche Anforderungen hinsichtlich Technik, Subjekt und Kontext müssen für die Implementierung einer solchen Intervention aus der Perspektive der Studierenden erfüllt sein?

Ziel ist es, die Einstellungsakzeptanz von primären Anti-Stress-Apps bei Studierenden mit Stressbelastungen im Studium zu analysieren und hieraus Anforderungen für die Implementierung digitaler Intervention im Kontext Hochschule abzuleiten.

## Methodik und Materialien

Die qualitative Studie wurde im Wintersemester 2020/2021 mit Studierenden der Universität Bielefeld durchgeführt. Zur Beantwortung der Fragestellungen wurde ein gestuftes Vorgehen mit Blick auf die theoriegeleitete Selektion von Interviewpartner*innen mittels eines quantitativen Fragebogens gewählt, das im Folgenden beschrieben wird.

### Rekrutierung der Stichprobe

An der Universität Bielefeld wurden im Zeitraum von Ende Oktober bis Mitte Februar Studierende der Universität Bielefeld über eine Informationskampagne rekrutiert. Die Teilnehmenden nahmen entlang einer im Vorfeld erfassten Selbstauskunft ein durch das Studium bedingtes Disstressempfinden wahr und befanden sich zu dem Zeitpunkt der Rekrutierung und späteren qualitativen Befragung nicht in einer psychotherapeutischen Behandlung.

Die Rekrutierung wurde durch die Studierendenvertretungen über E‑Mail-Verteiler der Fakultäten organisiert sowie die offiziellen Social Media Accounts bei Facebook und Instagram der Fachschaft für Gesundheitswissenschaften der Universität Bielefeld genutzt. Den Studieninteressenten wurden nach Kontaktaufnahme Informationsmaterialien und eine Einwilligungserklärung zugesandt. Nach der Einwilligung erfolgte in einem zweiten Schritt eine quantitative Abfrage anhand eines Kurzfragebogens. Dieser Fragebogen hatte zum Ziel, eine theoriegeleitete Auswahl von Teilnehmenden auf der Grundlage der UTAUT 2 zu ermöglichen sowie eine hohe Diversität in den für die Theorie relevanten Merkmalsausprägungen soziodemographischen Angaben zu erreichen [11]. Gleichwohl sollte damit ein individuelles Stressempfinden (Fragebogen zum Umgang mit Stress [SCI]; [6]) und die Technikaffinität (Fragebogen zur Technikaffinitäteinstellung und zu Umgang mit elektronischen Geräten) der Teilnehmenden erfasst werden [15].

Für die qualitative Befragung wurden daraufhin insgesamt 15 Studierende ausgewählt, die zuvor den Fragebogen ausgefüllt zurück gesendet haben. Die Charakteristika der Stichprobe wurden durch eine quantitative Erhebung mit Hilfe von Fragebögen erschlossen und sind in Tab. 1 genauer definiert.

**Tab. 1:** Stichprobencharakteristika

ID	Alter (Jahre)	Angestrebter Abschluss	Finanzierung	Vorerfahrung	Nutzung	Durchschnittliche Technikaffinität^e^	Durchschnittliches Stressempfinden^f^
*1*	22	BA^a^	B^c^	–	–	3,16	2,35
*2*	28	MA^b^	B, N^d^	✓	–	3,05	2,6
*3*	26	BA	B	✓	✓	2,89	2,78
*4*	31	BA	N	–	✓	2,79	2,5
*5*	30	MA	N	–	–	2,74	2,53
*6*	25	BA	N	✓	–	3,26	2,75
*7*	24	MA	B, N	–	✓	2,79	2,35
*8*	23	BA	N	–	✓	3	2,4
*9*	26	BA	N	✓	–	2,42	2,8
*10*	23	MA	N	–	✓	3,11	2,45
*11*	22	BA	N	–	✓	2,79	2,35
*12*	22	BA	N	–	–	2,84	2,26
*13*	22	BA	N	–	✓	3,12	2
*14*	25	MA	N	–	✓	1,95	2,6
*15*	22	BA	N	✓	–	3,05	2,65

^a^*BA* Bachelor

^b^*MA* Master

^c^*B* BAföG

^d^*N* Nebenbeschäftigung

^e^Mittelwert Technikaffinität (1 = trifft voll zu, 2 = trifft eher zu, 3 teils/teils, 4 trifft eher nicht zu, 5 = trifft gar nicht zu)

^f^Mittelwert Stressempfinden (1 = fast nie, 2 = manchmal, 3 = häufig, 4 = meistens)

Da die durchgeführte Studie mit *n* = 15 Teilnehmenden eine verhältnismäßig kleine Stichprobe umfasst, sind die in der Tabelle verausgabten Werte nicht repräsentativ adaptierbar, was allerdings auch nicht Anspruch eines im Kern qualitativen Studiendesigns sein kann. Die Tabelle dient insofern allein der Stichprobenbeschreibung.

### Leitfaden und Interviewdurchführung

Es wurden qualitative, halbstrukturierte und offene Interviews auf der Grundlage eines Leitfadens, welcher sich inhaltlich an der UTAUT 2 orientiert, durchgeführt. Die Theorie hilft, die treibenden Faktoren für die Nutzbarkeit und Akzeptanz von Technologie zu verstehen und darzustellen. Nach einer Erweiterung wurden in der vereinheitlichten Theorie 7 Schlüsselkonstrukte festgelegt, welche die Technologienutzung beeinflussen. Die Theorie definiert die Leistungserwartung als das Ausmaß, in dem die Nutzung einer Technologie den Nutzenden bei der Durchführung bestimmter Aktivitäten Vorteile bringt. Die Aufwandserwartung wurde als das Ausmaß an Leichtigkeit definiert, welches die Verbraucher mit der Nutzung von Technologie verbinden. Das Ausmaß, in dem Verbraucher wahrnehmen, dass Familienmitglieder oder das soziale Umfeld glauben, sie sollten eine bestimmte Technologie nutzen, wurde als sozialer Einfluss definiert. Erleichternde Bedingungen beziehen sich auf die Wahrnehmung der Verbraucher*innen hinsichtlich der Ressourcen und der Unterstützung, die für die Umsetzung eines Verhaltens zur Verfügung stehen. Das erweiterte UTAUT 2-Modell umfasst 3 weitere Determinanten: (1) die hedonische Motivation, die als Spaß oder Vergnügen bei der Nutzung einer Technologie definiert ist, (2) den Preiswert, der als kognitiver Kompromiss der Nutzenden zwischen den wahrgenommenen Vorteilen der Technologienutzung und den monetären Kosten definiert ist und (3) die Gewohnheit, die als das Ausmaß definiert ist, in dem Menschen dazu neigen, Verhaltensweisen aufgrund von Lernen automatisch auszuführen.

Der Leitfaden wurde innerhalb der Forschungsgruppe mehrfach gegenseitig überprüft und bei Bedarf entsprechend angepasst. Die Verständlichkeit der Fragen wurde anhand eines kognitiven Pre-Tests mit 3 Teilnehmenden getestet. Um ein einheitliches Verständnis sowie eine klare Bewertungsgrundlage für die qualitative Exploration der Nutzungsintention und Akzeptanz herzustellen, wurde ein kurzes Video entwickelt und in den Leitfaden und die Interviewsituation eingebunden. Das Video zeigte und erklärte den Teilnehmenden beispielhaft den Aufbau sowie die stressreduzierenden Elemente einer im Vorfeld ausgewählten Anwendung zur Distressreduktion und diente lediglich zu Demonstrationszwecken angelehnt an neutrale Reviewformate und um einen einheitlichen Konsensus hinsichtlich der App-Inhalte, Handhabung und Möglichkeiten zu schaffen. Da alle Inhalte, welche mit dem App-Hersteller in Verbindung gebracht werden könnten, unkenntlich gemacht wurden, diente das Video nicht jedoch zur Beeinflussung der Einschätzungen der Studienteilnehmenden gegenüber der App. Somit stand die Einhaltung von Objektivität bei der Videogestaltung im Vordergrund. Ein entsprechendes Skript zum Video wurde im Vorfeld erstellt und innerhalb der Forschungsgruppe gegenseitig überprüft. Gleiches gilt für den Videoaufbau. Über dies wurde die Videoerstellung medienpädagogisch begleitet. In der Konsequenz waren die Inhalte des Videos begrenzt auf die Vermittlung der reinen App-Funktionalität respektive der damit verbundenen Möglichkeiten zur Distressreduktion. Trotz des hohen Objektivitätsanspruchs können bei dieser Art der Intervention mediale Elemente der Beeinflussung nicht ausgeschlossen werden. Allerdings weisen die Ergebnisse der Studie darauf hin, dass von keiner beeinflussenden Wirkung im Sinne einer Bewerbung auszugehen ist.

Aufgrund der COVID-19-Pandemie wurden die Interviews online über eine Ende-zu-Ende verschlüsselte Videotelefoniesoftware in einem zeitlichen Rahmen von ca. 45 min geführt.

### Transkription und Auswertung

Damit eine Auswertung der Interviews im weiteren Verlauf der Forschung erfolgen konnte, wurden die Audioaufnahmen nach Dresing und Pehl (2017) in vereinfachter Form sowie wörtlich und nicht lautsprachlich transkribiert [3]. Nachfolgend wurden die Transkripte nach der strukturierten Inhaltsanalyse nach Mayring ausgewertet [17]. Maßgeblich für die Analyse waren die deduktiv aus der UTAUT 2 abgeleiteten Hauptkategorien, die induktiv anhand des Textmaterials erweitert wurden (Kategorien: Wissen bzw. Informiertheit, Ängste, stressassoziierte Aspekte und psychographische Aspekte). Die Ergebnisse der Interviews werden nach den Hauptkategorien „Leistungserwartungen“, „Aufwandserwartung“, „sozialer Einfluss“, „erleichternde Rahmenbedingungen“, „Preis-Leistungs-Verhältnis“, „Gewohnheit“ sowie „hedonistische Motivation“ eingeordnet. Zusätzlich ergab sich daraus die Subkategorie „Ängste“, welche einen Querschnitt der Hauptkategorien darstellt (Tab. 2).

**Tab. 2:** Ordnungskriterien und Kategoriendefinition der qualitativen Inhaltsanalyse

Kategorien	Definition
*Hauptkategorien*	
Leistungserwartung	Grad, zu dem ein Individuum davon ausgeht, dass die Nutzung einer Stress-App unterstützend wirken kann
Aufwandserwartung	Grad der Simplizität, der mit der Nutzung einer Stress-App verbunden wird (z. B. Integrierbarkeit in den Alltag, Benutzerfreundlichkeit)
Sozialer Einfluss	Grad der erwarteten Haltung und Einstellung im sozialen Umfeld und der Annahme, dass bedeutende Bezugspersonen der Ansicht sind, dass das Individuum eine Stress-App benutzen sollte (relevante Beziehungen sind dabei z. B. das unmittelbare soziale Umfeld)
Erleichternde Rahmenbedingungen	Grad, zu dem ein Individuum annimmt, dass eine technische oder organisationale Infrastruktur zur Verfügung stehen, die bei der Nutzung einer Stress-App unterstützend wirken
Preis-Leistung	Grad, zu dem ein Individuum bereit ist, Geld für die Funktionen einer Stress-App zu zahlen und welche Rolle das Finanzierungsmodell haben kann (Kaufpreis, In-App-Käufe oder Abo)
Gewohnheit	Grad der Habitualisierung eines Individuums im Sinne bereits vorherrschender Handlungs- bzw. Nutzungsgewohnheiten
Hedonistische Motivation	Grad, zu dem ein Individuum die Stress-App einsetzt, um positive Emotionen zu erlangen und negative Emotionen zu vermeiden
*Subkategorien*	
Ängste	Grad, zu dem ein Individuum Bedenken bei der Nutzung von Stress-Apps hat (z. B. hinsichtlich einer befürchteten Ungewissheit bei der Datensicherheit)

## Ergebnisse

Die Ergebnisse aller anhand der UTAUT 2 analysierten Kategorien werden im Folgenden detailliert erläutert und zusammenfassende Aussagen aus den Interviews dargestellt.

### Ergebnisse der Leistungserwartungen

Die teilnehmenden Studierenden erwarten von einer App zur Stressreduktion insbesondere Linderung des wahrgenommenen Leidensdruckes aufgrund von Stress in Akutsituationen. Langfristig sollte eine App zu einem konstruktiveren Umgang mit Stress anleiten und sich nachweislich positiv auf psychische und körperliche Belastungen auswirken. Aus der Perspektive der Befragten wird dabei die Funktionalität der Dokumentation der eigenen Fortschritte und die Schaffung von motivationalen Anreizen zur Nutzung besonders hervorgehoben, um die Nachhaltigkeit ihrer Wirkung zu fördern: „Von einer Anti-Stress-App allgemein würde ich erwarten, dass sie mir helfen mit dem Stress, den ich habe, umzugehen und mir Kompetenzen zur Stressbewältigung nahebringt. Außerdem erwarte ich noch, dass mir meine Entwicklung visualisiert wird.“ (I3 Z45).

### Ergebnisse der Aufwandserwartungen

In der Betrachtung der artikulierten Aufwandserwartungen wird deutlich, dass eine nicht leicht zugänglich und zielgruppengerecht gestaltete App ebenso als belastend respektive als Stressor wahrgenommen werden kann. Förderlich wirke sich dabei ein hohes Maß an Individualisierbarkeit aus. Ebenso besteht die Erwartung, dass sich eine App einfach in die Tagesabläufe integrieren lassen sollte – auch damit eine zeitliche Nähe zu den erlebten, stressinduzierenden Situationen besteht. Ein strukturiertes, ansprechendes „user interface design“ (UI) soll durch multimediale Reize in Bild und Ton ergänzt werden. Auch „gamification“ (engl. von „game“: „Spiel“), also Spielelemente, wird hierbei als weiteres didaktisches Element gesehen, welches initiale Aufwandserwartungen reduzieren kann: „Das Design sollte intuitiv und einfach zu bedienen sein und mich persönlich direkt ansprechen.“ (I5 Z163).

### Ergebnisse der sozialen Einflüsse

Die Einstellung des sozialen Umfelds bezüglich der eigenen Nutzungsintention oder Nutzung einer App zur Stressreduktion wird nicht als relevant oder einflussgebend für das eigene Nutzungsverhalten interpretiert. Zu unterscheiden sind hiervon soziale Empfehlungsstrukturen (Befreundete, Verwandte oder Lehrende) sowie bereits gesammelte Erfahrungswerte anderer, die wahrgenommen oder explizit recherchiert und bei der Auswahl und Entscheidungsfindung bezüglich der eigenen Nutzung berücksichtigt werden. Auch digitale Bewertungen anderer Nutzenden und Rezensionen in App- und Playstores nehmen Einfluss auf die Kauf- oder Downloadentscheidung. Werbeanzeigen und Blogs im Internet (z. B. präsentiert von Influencer*innen) werden aus der Perspektive von Studierenden als Orientierungshilfe für die Auswahl einer App wahrgenommen. Wissenschaftliche Veröffentlichungen sind für Studierende prinzipiell eine wichtige Quelle, werden aber mit Blick auf den Entscheidungsprozess der App-Nutzung weniger stark wahrgenommen: „Wenn sie [die App] mir von Freunden oder im Internet empfohlen wird, dann hat das einen positiven Einfluss auf mich, weil ich dann weiß, dass sie Anderen schon geholfen hat.“ (I5 Z45).

### Ergebnisse der nutzungserleichternden Rahmenbedingungen

Die Bewertungsmechanismen von nutzungserleichternden Rahmenbedingungen von Apps zur Stressreduktion können umwelt-, objekt- und subjektbezogen definiert werden. Diesbezüglich sehen Studierende insbesondere die Finanzierung durch Krankenversicherungen (KVen) oder Lizenzen für Hochschulen als erleichternde umweltbezogene Rahmenbedingung an. Objektbezogene Rahmenbedingungen beziehen sich bspw. auf das optische Design (UI), die Struktur, die Handhabung und Gebrauchstauglichkeit (engl. auch „user experience“, UX) der App. An dieser Stelle artikulieren die Studierenden ein hohes Maß an Individualisierbarkeit der stressreduzierenden Maßnahmen innerhalb des Angebots. Die eigene Motivation zur Nutzung und die individuelle Nutzungsbereitschaft im Alltag können den subjektbezogenen handlungserleichternden Rahmenbedingungen zugeordnet werden. Hierzu zählt auch das persönlich empfundene Stressniveau, was ggf. einen Handlungsdruck auslöst und die Nutzungsintention beeinflusst: „Gut wäre, wenn so eine App komplett barrierefrei wäre und in verschiedenen Sprachen abrufbar ist. Also nicht nur auf Deutsch oder Englisch ist, sondern z. B. auch auf Französisch, Türkisch, Russisch etc. Und wenn es dann noch kostenlose Lizenzen für Studierende gibt – umso besser!“ (I8 Z2013)*.*

### Ergebnisse der Preis-Leistungs-Erwartung

Kostenlose Anwendungen werden grundsätzlich gegenüber kostenpflichtigen Apps bevorzugt, wenn diese vergleichbare Inhalte bieten. Ein Kauf wird erst dann in Erwägung gezogen, wenn die App in einer kostenfreien Testphase ausgiebig inspiziert und ein persönlicher Nutzen festgestellt werden konnte. Die Kaufbereitschaft bleibt selbst nach persönlich festgestellter Wirksamkeit eingeschränkt. Dennoch sind Studierende dann bereit, monatliche Kosten für die Anwendung in Kauf zu nehmen, wenn ein subjektiver, gesundheitlich relevanter Nutzen festgestellt werden konnte. Die Nutzungsbereitschaft steigt unter Studierenden, wenn Apps von seriösen Kooperationspartner*innen wie Hochschulen oder KVen kostenlose Nutzungslizenzen erhalten: „Wenn ich wirklich in diesem kostenlosen Zeitraum festgestellt habe, dass die App mir total den Mehrwert bietet, was auch keine andere App jetzt so abdecken kann, dann wäre ich schon bereit monatlich dafür Geld auszugeben.“ (I10 Z73).

### Ergebnisse der Gewohnheit

Die Befragten gaben an, unterschiedliche individuelle Methoden zur Stressreduktion zu nutzen. Darunter Sport, Ausgleich durch soziale Kontakte, Planung und Organisation des Alltags und auditive Einschlafhilfen. Diese werden nur von einem Teil der Befragten (*n* = 8) auf mobilen Endgeräten genutzt, da die Kompatibilität und Funktionalität des Mediums zu diesem Zweck in Frage gestellt werden. Auf eine digitale Anwendung zur expliziten Stressreduktion wird dann zugegriffen, wenn bisherige Bewältigungsstrategien nicht mehr wirksam oder (z. B. durch die COVID-19-Pandemie) verfügbar sind. Die App ist bequemer und schneller anwendbar als vergleichbare ortsgebundene Angebote wie Meditationskurse. Diese Leistungserwartung korreliert mit der Gewohnheit der Nutzenden, da sie das Potenzial begünstigt, akut in Stresssituationen mit Hilfe der App zu intervenieren. Es wird also gefordert, bestmöglich auf die Gewohnheit der Nutzenden eingehen zu können, um die Anwendung effektiver auf akute Situationen und individuelle Bedürfnisse auszurichten: „Sie [die App] muss auf jeden Fall leicht in den Alltag zu integrieren sein, damit man da nicht noch so viel reininvestieren muss, bis sich der Stress reduziert, wie wenn ich eine halbe Stunde zu meinem Kurs fahre. Ich kann so spontan in Stresssituationen mit der App wieder runterkommen.“ (I7 Z45).

### Ergebnisse der hedonistischen Motivation

Damit die Motivation zur Nutzung der Anti-Stress-App gesteigert wird, wünschen sich die Teilnehmenden die Dokumentation ihres Leistungsfortschritts sowie Anreize, die zur Anwendung auffordern. Push-Benachrichtigungen oder die Sichtbarkeit der App auf dem Startbildschirm werden dabei besonders motivierend hervorgehoben, gleichzeitig aber auch als Stressoren bewertet. Ebenso wird deutlich, dass im Sinne einer reflexiven Nutzung mobiler Endgeräte, die summative Bildschirmzeit als ein kritischer Faktor wahrgenommen wird. Die ständige Expansion dieser durch zusätzliche Apps steht im Gegensatz zu dem Hilfebedürfnis im Rahmen der Bewältigung von Stresssituationen: „Ich will meine generelle Bildschirmzeit reduzieren und eine App, die auf einem mobilen Endgerät läuft, ist praktisch. Allerdings ist es für mich schwer mein Vorhaben umzusetzen, denn so würde ich noch mehr Zeit am Handy verbringen.“ (I7 Z36).

Die Präventions- und Interventionsmaßnahme, welcher die App zugrunde liegt, ist nachhaltig motivierend, wenn ein Kurs aus mehreren Einheiten besteht dessen Anwendungsdauer individualisierbar ist. Darüber hinaus beeinflussen Sprecher*innenstimmen auditiver Elemente, die Entscheidung, die Maßnahme erneut oder bis zum Ende durchzuführen.

### Ergebnisse der Ängste

Die Subkategorie „Ängste“ stellt Sorgen und Bedenken der Studierenden im Querschnitt zu allen Hauptkategorien dar und wurde induktiv gebildet. Dabei zeigen sich insbesondere Bedenken mit Blick auf den Datenschutz, der Wirksamkeit der Interventionsmaßnahme sowie der allgemeinen Qualität der App. Darüber hinaus äußerten sich die Befragten kritisch gegenüber der Ausweitung der Abhängigkeit von Technologien im Rahmen von Problemlösungsstrategien. Die autonome Anwendung einer App, welche von unter Stress leidenden Studierenden genutzt wird, könnte unter Einfluss persönlicher Determinanten negative Einflüsse auf das Stresserleben haben: „Die App sollte nicht nur seriös wirken, sondern es auch sein. Datenschutz ist dabei auch total wichtig! Nicht, dass meine gesundheitsbezogenen Daten an irgendwelche Drittanbieter weitergegeben werden.“ (I11 Z136).

## Diskussion

Wie die aktuelle Forschung bestätigt [13, 14], verdeutlichen die Ergebnisse dieser Studie, dass Studierende durch bereits gesammelte, positive Erfahrung mit Anti-Stress-Apps oder unter gewissen Bedingungen (z. B. Leidensdruck oder Neugier) bereit sind, Apps zur Stressbewältigung zu nutzen. Je nach Vorerfahrungen kann dies sowohl hemmend wie auch fördernd wirken. Dabei geht jedoch die Akzeptanz von E‑Mental-Health-Angeboten nicht mit der Häufigkeit der Nutzung einher, genauso wie die Technikaffinität nicht in direktem Bezug zum Interesse an technisch gestützten Interventionen steht.

Die Ergebnisse der Leistungs- und Aufwandserwartungen sowie jene der Rahmenbedingungen geben Aufschluss über die Komplexität einer stressreduzierenden App: Die Vielzahl der genannten Anforderungen an die technische Umsetzung verdeutlicht die *Komplexität* des Umfangs im Bereich Softwareengineering und UI/UX-Design, da Bedürfnisse sehr individuell und unterschiedlich sind, zu dessen Ergebnis auch Löcherer und Apolinário-Hagen (2017) in ihrer Erhebung kamen. Daraus bilden sich jedoch bedeutende Stärken digitaler Anwendungen gegenüber gewohnten Methoden zur Stressbewältigung Studierender, wie der geringe Aufwandseinsatz in Bezug auf den zeit- und ortsunabhängigen Gebrauch und die hohe Individualisierbarkeit. Zudem wird ein hohes Maß an Privatsphäre ermöglicht, sodass die Übungseinheiten im selbstgewählten Umfeld durchgeführt werden können, was gerade bei einem sensiblen Thema wie Stressbelastung von Vorteil sein kann. Barrieren, wie die Inanspruchnahme von Hilfsangebote durch Dritte (z. B. Ärzt*innen oder Therapeut*innen) können durch die digitalen Anwendungen sogar umgangen werden [4, 14, 16]. Anti-Stress-Apps können so einen Beitrag leisten, Behandlungsdefizite auszumerzen und Versorgungsspielräume auszuweiten [15, 21]*. *Werden *Nutzungslizenzen* durch die genannten Leistungserbringer*innen kostenfrei zur Verfügung gestellt, erhöht dies die Nutzungsbereitschaft maßgeblich.

Bei den Interviews zeigte sich eine Diskontinuität hinsichtlich der Anwendung von stressreduzierenden Maßnahmen. Seltener waren eine langfristige Stressreduktion und somit die Prävention psychischer Erkrankungen das Ziel. Vielmehr wurde eine gezielte Hilfe bspw. in der Prüfungsphase oder akuten Stresssituationen gewünscht, da insbesondere der Eingriff einer derartigen App durch regelmäßiges Nutzen in das Alltagsgeschehen ambivalent durch die Befragten gesehen wurde. Diese Erkenntnis stützt die Aussage, dass die Anwendung der App manche Teilnehmende mehr belastet, als das empfundene Stressgefühl zu reduzieren. Hier ist (durch weitere Forschung mit einer größeren, diverseren Stichprobe) zu klären, für welche Personengruppe dies konkret zutrifft und welche Maßnahmen für jene stressreduzierend wirksam sind.

Eine transparente Datenschutzpolitik ist bei wenigen Befragten ausschlaggebend für die Nutzung, für andere ist der Datenschutz eher irrelevant und stellt somit keine einflussgebende Nutzungsbedingung für das Nutzungsverhalten dar. Hier besteht eine hohe Disparität, welche an dieser Stelle ungeklärt bleibt. Dennoch ist dies ein wesentlicher Faktor bei der Etablierung einer Anwendung mit Eingriff in die Alltagsplanung, welcher individuell gewünscht wie unerwünscht sein kann.

Die Meinungen der Befragten über mögliche Optionen von Anwendungen auf mobilen Endgeräten sind konträr*. *Erinnerungen durch *Push-Benachrichtigungen*, die zur Nutzung der Maßnahme auffordern, können in Abhängigkeit zur subjektiven Wahrnehmung motivierend oder stressinduzierend wirken, was bisherige Forschung unterstreicht [11, 12]. Ebenso ist die Sichtbarkeit der App auf dem Startbildschirm und das Eingreifen in den persönlichen Kalender unter den Befragten zu gleichen Teilen gewünscht wie unerwünscht. Wichtig war allen Befragten, dass die Anwendung fordernd aber nicht überfordernd wirkt, was anhand der Ergebnisse der Subkategorie unterstrichen wird.

### Limitationen

Die zentralen Limitationen dieser qualitativen Studie beziehen sich auf das methodische Vorgehen in der Rekrutierung, der daraus resultierenden Stichprobe und den Studienzeitraum. Es ist davon auszugehen, dass ein Großteil der Teilnehmenden Studierende der Fakultät für Gesundheitswissenschaften sind, da verstärkt über die Kommunikationskanäle dieser Fakultät rekrutiert wurde. Darüber hinaus wurde die Erhebung während der COVID-19-Pandemie durchgeführt, wobei das zuvor festgelegte Studiendesign an die pandemisch bedingten Regeln kurzfristig angepasst werden musste. Daher wären hinsichtlich des Studiendesigns folgende Änderungen notwendig, um die Aussagekraft der Studienergebnisse weiter zu erhöhen und offene Diskussionspunkte zu betrachten:

- Vergrößerung des Untersuchungszeitraums, um eine Testphase durchführen zu können, in der die Studierenden eine Anti-Stress-App eigenständig testen können.
- Ausweitung und Vergrößerung der Stichprobe auf mehr Hochschulen und Fakultäten sowie eine repräsentative Abbildung geschlechtlicher Identitäten und Studienabschlüsse.

Unter den pandemischen Einflüssen kann durch das hohe Maß an Veränderung der Lebenssituation Studierender ein erhöhtes Stressempfinden vermutet werden. Dies könnte die Einstellungsakzeptanz zu Apps als digitale Alternative zu gewohnten Anwendungen zur Stressbewältigung allgemein begünstigen. Dennoch war die Pandemie nicht ausschlaggebender motivierender Faktor, die Studie durchzuführen. Zum Zeitpunkt der Erhebung waren die Auswirkungen der Pandemie auf die Studierenden noch nicht abzusehen und eher vage zu vermuten. Darüber hinaus gab es noch keine Forschungsgrundlage dazu. Nicht zuletzt sei darauf hingewiesen, dass bei der Erstellung des Leitfadens keine externe Überprüfung dessen initiiert wurde.

## Ausblick

Die Ergebnisse der Studie zeigen viele Potenziale in der Nutzung primärer Anti-Stress-Apps als besonders niederschwellige Intervention zur Stressreduktion auf, welche ebenfalls durch bisherige Forschung bestätigt werden können. Die Potenziale dieser Anwendungen gehen jedoch mit einigen Herausforderungen hinsichtlich der Einstellungsakzeptanz einher, welche sich besonders auf Ambivalenzen, Skepsis und Ängste der Studierenden bezieht. Es wird nur dann eine persönliche Wirksamkeit festgestellt, wenn die App stark auf individuelle Bedürfnisse zur Anwendung eingeht. Mittels der UTAUT 2-Theorie konnten dahingehend diese differenzierten Nutzungsbedingungen identifiziert werden. Ob diese kritische Haltung der Studierenden bezüglich der Integration in den Alltag in Korrelation mit einem hohen Maß an Symptomen psychischer Erkrankungen unter Studierenden steht, kann an dieser Stelle nur vermutet werden und bedarf weiterer Klärung. Ein ausschlaggebender Anreiz, eine solche App zu nutzen, ist die Kostenübernahme durch die KVen oder Hochschulen. Inwiefern das Studium dadurch mit deutlich weniger Belastungen absolviert werden oder diese präventiv vermeiden könnte, ist in weiterer Forschung zu klären. So würde u. a. ggf. ein Vorteil für die entsprechenden Hochschulen entstehen, den eigenen Attraktivitätsgrad zu steigern.

Da es sich bei Stress um eine subjektive Empfindung handelt, bedarf es entsprechend einer individuellen Herangehensweise zur Linderung. An dieser Stelle ist in einer größeren Befragung zu klären, welche stressreduzierenden Optionen und Merkmale in einer App vorhanden sein müssen. Die komplexen Herausforderungen an die technischen Hintergründe plädieren für das Einbeziehen von Public-Health-Expertise in den (Software‑)Entwicklungsprozess, damit die gesundheitsfördernden Aspekte ausreichend bedient werden und so die Wirksamkeit der Anwendung gesteigert werden kann. Durch einen interdisziplinär aufgestellten Hintergrund und genannte Kooperationspartner*innen steigt das Vertrauen der Nutzenden in die App und wird dadurch positiv konnotiert. Aufklärungs- und Marketingkampagnen sollten Vorteile einer bestimmten App aufzeigen, um Betroffene für die Anwendung und Nutzung zu überzeugen.

Anhand der aufgeführten Gesichtspunkte ist erkennbar, dass das Angebot stressreduzierender Interventionsmaßnahmen durch ein digitales Angebot von Anti-Stress-Apps Defizite der Versorgung wirksam ausgleichen kann. Ausschlaggebend dafür sind besonders die niederschwelligen Anreize, welche durch eine vielfältige Produktpalette innerhalb einer App abgedeckt werden könnten und durch hohe Individualisierbarkeit ein hohes Potenzial darstellen, psychische Belastungen zu minimieren, indem sie bedürfnisorientiert auf die Nutzenden eingehen. Dem entgegen stehen die damit einhergehenden Kosten für den interdisziplinären Ausbau entsprechender Softwareentwicklung und die dadurch bedingt hohen Endkosten für die Nutzenden. Da die Einstellungsakzeptanz von Anti-Stress-Apps grundsätzlich bestätigt werden konnte, ist ein entsprechend hohes Nutzungsverhalten zu erwarten, wenn vorhandene Angebote differenzierter ausgebaut werden.

## Fazit für die Praxis

- Anti-Stress-Apps sollten für Studierende aufgrund der außerordentlichen Belastungen kostenlos sein oder von Kostenträger*innen (Hochschulen oder KVen) in der Endnutzung sowie der Entwicklung (mit)finanziert werden.
- Vorteile des Zusammenschlusses mehrerer stressreduzierender Optionen sind hervorzuheben und eine Marke zu etablieren, die synonym mit Entspannung, Stressreduktion und einer besseren Gesundheit konnotiert wird, um eine Brücke zwischen Akzeptanz und Nutzung zu schlagen.
- In der Softwareentwicklung sollten ein hohes Maß an bedürfnisorientierter Individualisierbarkeit im UI (optisches Design)/UX („user experience“)-Design, zur Steigerung der Motivation und der subjektiven Wirksamkeit berücksichtigt werden.
- In weiterführender Forschung sollte Studienteilnehmenden der selbstständige Umgang mit Apps in einer mehrwöchigen Testphase ermöglicht werden, um differenzierte Herangehensweisen zu beobachten und ein weites Feld an Nutzungsbedingungen, -verhalten und Akzeptanz in der Praxis zu untersuchen.
